# Insight into Grain Refinement Mechanisms of WC Cemented Carbide with Al_0.5_CoCrFeNiTi_0.5_ Binder

**DOI:** 10.3390/ma17174223

**Published:** 2024-08-27

**Authors:** Fengming Qiang, Pengfei Zheng, Pan He, Wen Wang, Ying Zhang, Peng Han, Kuaishe Wang

**Affiliations:** 1 School of Metallurgical Engineering, National and Local Joint Engineering Research Center for Functional Materials Processing, Xi’an University of Architecture and Technology, Xi’an 710055, China; qiangfengming@xauat.edu.cn (F.Q.); 18792320467@163.com (P.Z.); 18165116317@163.com (P.H.); zy2104211081@163.com (Y.Z.); hanpeng2021@126.com (P.H.); 2 Xinjiang Xiangrun New Material Technology Co., Ltd., Xi’an 710000, China

**Keywords:** cemented carbide, high entropy alloy binder, hot press sintering, grain refinement

## Abstract

High-entropy alloys (HEA) as a kind of new binder for cemented carbide have garnered significant attention. In this work, WC/(17~25 wt.%)Al_0.5_CoCrFeNiTi_0.5_ cemented carbides were prepared by hot pressing sintering (HPS), and the reactions between WC powder and Al_0.5_CoCrFeNiTi_0.5_ powder during hot pressing sintering were elucidated. It found that different from traditional Co binder, the Al_0.5_CoCrFeNiTi_0.5_ binder effectively inhibited WC grain growth. During HPS, the decomposed W and C atoms from WC diffused into the Al_0.5_CoCrFeNiTi_0.5_ binder, reacted with the elements in the binder, and then formed the M(Co, Fe, Ni)_3_W_3_C phase. The back-diffusion of W and C atoms to WC grains was restricted by the Al_0.5_CoCrFeNiTi_0.5_ alloy and inhibited them from re-precipitating onto the large undissolved WC grains. As a result, the average size of WC grains in the cemented carbides was less than 200 nm. This work bright new insight into the grain refinement mechanisms of WC cemented carbide with HEA binder and provide a guidance for designing performance-stable WC/HEA cemented carbide and promoting their application.

## 1. Introduction

Cemented carbide is a kind of composite that is prepared by powder metallurgy with refractory metal as matrix and ductile metal as a binder. The cemented carbide has high toughness, heat and wear resistance, and are widely used as cutting tools and wear parts. WC/Co cemented carbide is one of the widely used cemented carbides, which is prepared with WC as the hard phase and Co as the binder. However, Co, as a toxic strategic resource, has a high application cost. Besides, WC/Co cemented carbide prepared by Co as binder has poor high temperature softening resistance, resulting in the degradation in the hardness, corrosion/oxidation resistance of materials and a reduced service life [1,2]. Therefore, the development of new binders with excellent performance and low cost is a research hotspot in the field of cemented carbide in recent years.

High entropy alloy (HEA) possesses excellent properties and has good wettability with WC, which sheds new light on the development of new binders and breaks the conventional concept of the single-element binder [3,4,5]. Luo et al. [4] prepared WC/AlCoCrCuFeNi cemented carbide by discharge plasma sintering technique, and the microhardness and the fracture toughness K_IC_ value were increased by 43% and 12.5% respectively compared with the conventional WC/Co cemented carbide. Chen et al. [5] prepared fine-grained WC/Al_0.5_CoCrCuFeNi cemented carbide using vacuum hot-press sintering, whose hardness was 200~300 HV higher than that of WC/Co cemented carbide at both room temperature and high temperature. It is obviously that the HEA binder can improve the mechanical properties of cemented carbides compared with the Co binder. Nevertheless, the feasibility of HEAs as a binder should be addressed by digging out the detailed reaction mechanism between WC and HEA during fabrication, for designing performance-stable WC/HEA cemented carbide and promoting their application.

The CoCrFeNi-based HEA is the most commonly used binder [6,7], as the Co, Cr, Fe, and Ni belong to the same periodic element group, possessing comparable atomic sizes and electronegativities, and is easy to form a simple solid solution. Usually, one or two additional elements will be incorporated to achieve the desired properties. Especially, the addition of Al and Ti elements can improve the high-temperature oxidation resistance, corrosion resistance and wear resistance of CoCrFeNi-based HEA. However, excessive aluminum addition will reduce the wettability between WC and HEA, leading to a deterioration in fracture toughness [3,4]. Besides, excessive titanium content in AlCoCrFeNiTi*_x_* can lead to the formation of Laves phase, which increases the brittleness of the HEA [8]. Thus, in this work, the Al_0.5_CoCrFeNiTi_0.5_ is selected as a binder to prepare the WC/Al_0.5_CoCrFeNiTi_0.5_ cemented carbide by vacuum hot pressing sintering technique. The reactions between WC and HEA during hot pressing sintering (HPS) and the effect mechanism of Al_0.5_CoCrFeNiTi_0.5_ binder on the microstructure characteristics of WC cemented carbide are investigated, aiming to provide theoretical guidance for the development of new WC/HEA cemented carbide.

## 2. Experimental Materials and Methods

**Figure 1:**
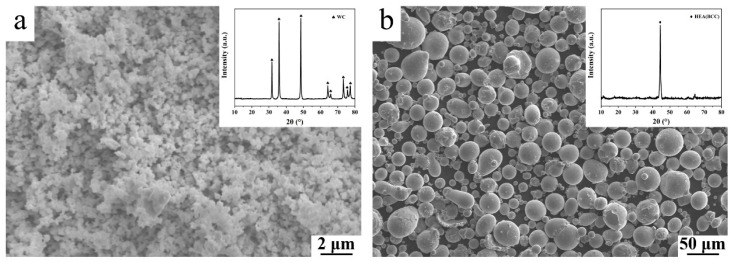
SEM images and XRD patterns of (**a**) WC powders and (**b**) Al_0.5_CoCrFeNiTi_0.5_ powders.

WC powders and Al_0.5_CoCrFeNiTi_0.5_ powders are selected as the raw material. The WC powders are irregular and agglomerated with the particle size of around 200 nm (Figure 1a) and a purity of more than 99.9%. The Al_0.5_CoCrFeNiTi_0.5_ powders show spherical morphology with the particle size specification of 15~53 μm (>90 vol.%) and exhibits a body-centered cubic (BCC) structure (Figure 1b). The real chemical composition of the Al_0.5_CoCrFeNiTi_0.5_ powders is shown in Table 1.

The Al_0.5_CoCrFeNiTi_0.5_ powders were mixed with the WC powders in the proportions of 17 wt.%, 20 wt.% and 25 wt.%. The carbide ball mill jars and grinding balls were used to grind the mixed powders for 20 h with a ball-to-powder ratio of 5:1. The dispersant was 2 % anhydrous ethanol, and the grinding speed was 200 rpm. The mixed powders were dried and then sieved using a 500-mesh screen. Sintering was performed on a vacuum hot pressing sintering system (ZT-110-20Y, Shanghai Chenhua Science Technology Co., Ltd., Shanghai, China), with a sintering temperature of 1250 °C, a holding time of 240 min, a sintering pressure of 30 MPa, and a heating rate of 7 °C/min.

The phase constituents of the samples at different conditions were analyzed by an X-ray diffractometer (XRD, D8 ADVANCE A25, Bruker, Karlsruhe, Germany) using a Cu Kα radiation with an angle range of 20–80°, and the voltage, current and scanning step were 40 kV, 50 mA and 0.02°/s, respectively. The microstructures of the WC/Al_0.5_CoCrFeNiTi_0.5_ cemented carbide samples was analyzed by scanning electron microscope (Gemini SEM 300, Zeiss, Oberkochen, Germany) equipped with an energy dispersive spectrum (EDS) and electron backscattered diffraction (EBSD) camera. The mechanically polished surfaces of the samples were then vibratory grinded in colloidal silica suspension for 4 h to yield a stress-free surface for EBSD images.

## 3. Results and Discussion

### 3.1. Formation of Carbides in HPSed WC/HEA

**Figure 2:**
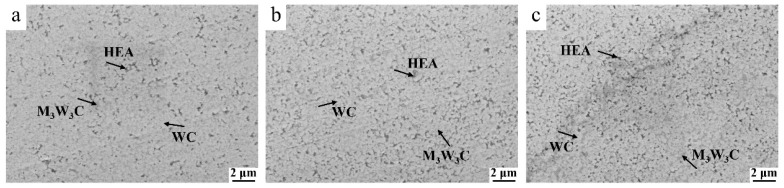
SEM images of the HPSed WC/Al_0.5_CoCrFeNiTi_0.5_ cemented carbide: (**a**) 17 wt.% Al_0.5_CoCrFeNiTi_0.5_; (**b**) 20 wt.% Al_0.5_CoCrFeNiTi_0.5_; (**c**) 25 wt.% Al_0.5_CoCrFeNiTi_0.5_.

Figure 2 displays the SEM images of the WC/Al_0.5_CoCrFeNiTi_0.5_ cemented carbides. It can be seen that the cemented carbides with different contents of binder are relatively dense and without obvious pores. The WC phase and HEA phase can be clearly identified as light and dark color by the contrast, and the Al_0.5_CoCrFeNiTi_0.5_ binder is distributed evenly around the WC grains. That is because, on one hand, a layer of Al_0.5_CoCrFeNiTi_0.5_ thin film has been formed on the surface of WC powders due to severe plastic deformation during the ball milling process. On the other hand, under the action of pressure and capillary force during HPS, the Al_0.5_CoCrFeNiTi_0.5_ binder undergoes plastic flow and diffuses into the gap between the WC particles, gradually filling or reducing the gap during the interdiffusion with W and C atoms [9].

**Figure 3:**
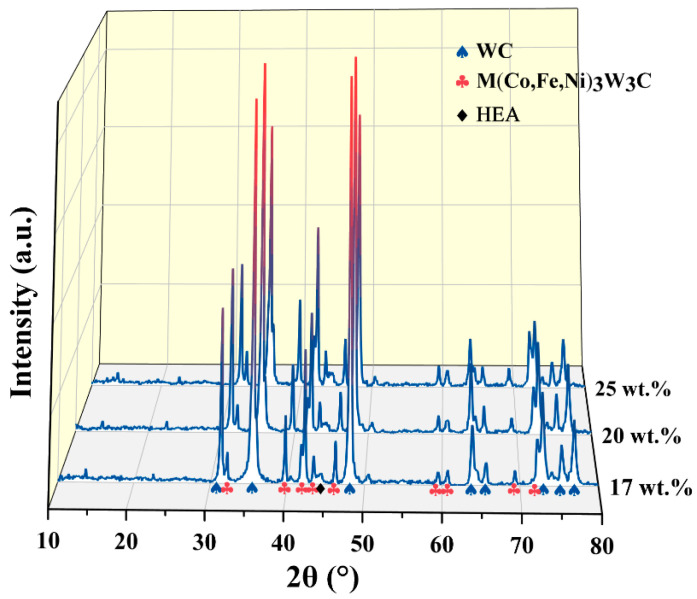
XRD patterns of HPSed WC/HEA cemented carbides with different content of Al_0.5_CoCrFeNiTi_0.5_ binder.

It should be mentioned that apart from the bright WC phase and dark Al_0.5_CoCrFeNiTi_0.5_ phase, a grey phase can be observed in the SEM images. That is M(Co,Fe,Ni)_3_W_3_C phase, as the XRD patterns of the HPSed WC/Al_0.5_CoCrFeNiTi_0.5_ cemented carbides displayed in Figure 3. This phase is formed by the mutual diffusion of W and C atoms in WC and the atoms in the Al_0.5_CoCrFeNiTi_0.5_ binder during the HPS [10,11,12]. The M(Co,Fe,Ni)_3_W_3_C phase is similar to the Co_3_W_3_C phase in traditional WC/Co cemented carbide [13]. By comparing the XRD patterns of cemented carbides with different Al_0.5_CoCrFeNiTi_0.5_ contents, it can be found that the intensity of the M(Co,Fe,Ni)_3_W_3_C diffraction peaks (41.9° and 72.5°) increases significantly with the increase of Al_0.5_CoCrFeNiTi_0.5_ binder content. This is resulted from the formation mechanism of M(Co,Fe,Ni)_3_W_3_C phase. The more content of the increase of Al_0.5_CoCrFeNiTi_0.5_ binder, more atoms in Al_0.5_CoCrFeNiTi_0.5_ could react with the W and C atoms to form the M(Co,Fe,Ni)_3_W_3_C phase, thereby increasing the content of M(Co,Fe,Ni)_3_W_3_C phase.

**Figure 4:**
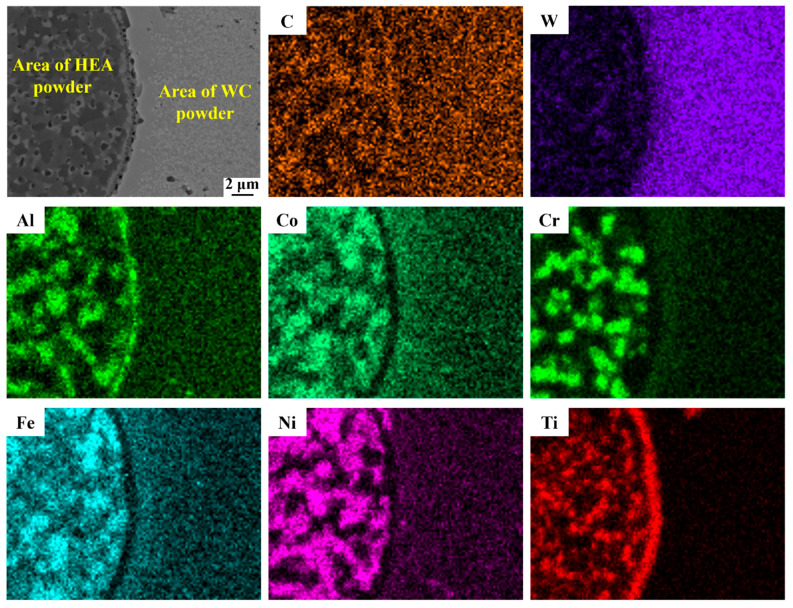
EDS images of HPSed WC/Al_0.5_CoCrFeNiTi_0.5_ cemented carbide.

To further understand the reactions between WC and HEA, the EDS composition analysis was conducted in an unfully-diffusion area with the interface of WC powder and Al_0.5_CoCrFeNiTi_0.5_ binder, as shown in Figure 4. It can be observed that the C element is almost uniformly dispersed in the WC region and the Al_0.5_CoCrFeNiTi_0.5_ binder region, while the W element is mainly distributed in the WC region and there still exists some W element in the Al_0.5_CoCrFeNiTi_0.5_ binder region. It indicates that although the diffusion rate of W atom is much slower than that of C atom, the decomposition and diffusion of WC has happened during HPS. Similarly, because of atom diffusion and migration, vacancies occur in the interior of the Al_0.5_CoCrFeNiTi_0.5_ binder, and some elements are enriched, as seen from the element distribution maps in Figure 4. The W and C atoms that diffused into the Al_0.5_CoCrFeNiTi_0.5_ binder prefer to aggregate and react with some elements of Al_0.5_CoCrFeNiTi_0.5_ HEA to form the M(Co,Fe,Ni)_3_W_3_C phase. The processes of WC dissolution and the M(Co,Fe,Ni)_3_W_3_C phase formation can be illustrated by three steps shown in Equations (1)–(3) [14].

2WC = W_2_C + C (1)

W_2_C = 2W + C (2)

WC + 2W + 3M = M_3_W_3_C (3)

It should be mentioned that the Cr and Ti atoms in Al_0.5_CoCrFeNiTi_0.5_ HEA is easy to react with C atoms which consumed some C atoms, resulting in there are some retained W atoms. As the Co, Fe, and Ni atoms have almost equal atomic radii ($r_{Co}$ = 1.25 Å; $r_{Cr}$ = 1.26 Å; $r_{Fe}=$ 1.25 Å; $r_{Ni}$ = 1.24 Å) and electronegativity ($E_{Co}$ = 1.88, $E_{Cr}$ = 1.66, $E_{Fe}$ = 1.91; $E_{Ni}$ = 1.83), which allows them to dissolve into each other and reacted with the retained W atoms and WC, leading to the formation of the same structured M(Co,Fe,Ni)_3_W_3_C phase. Here, the Al atom has relatively large atomic radius making it difficult to be dissolved. Thus, the M(Co,Fe,Ni)_3_W_3_C phase is not a traditional carbide but actually a carbon-deficient phase [12,13].

### 3.2. Mechanism of WC Grain Size Evolution in HPSed WC/HEA

**Figure 5:**
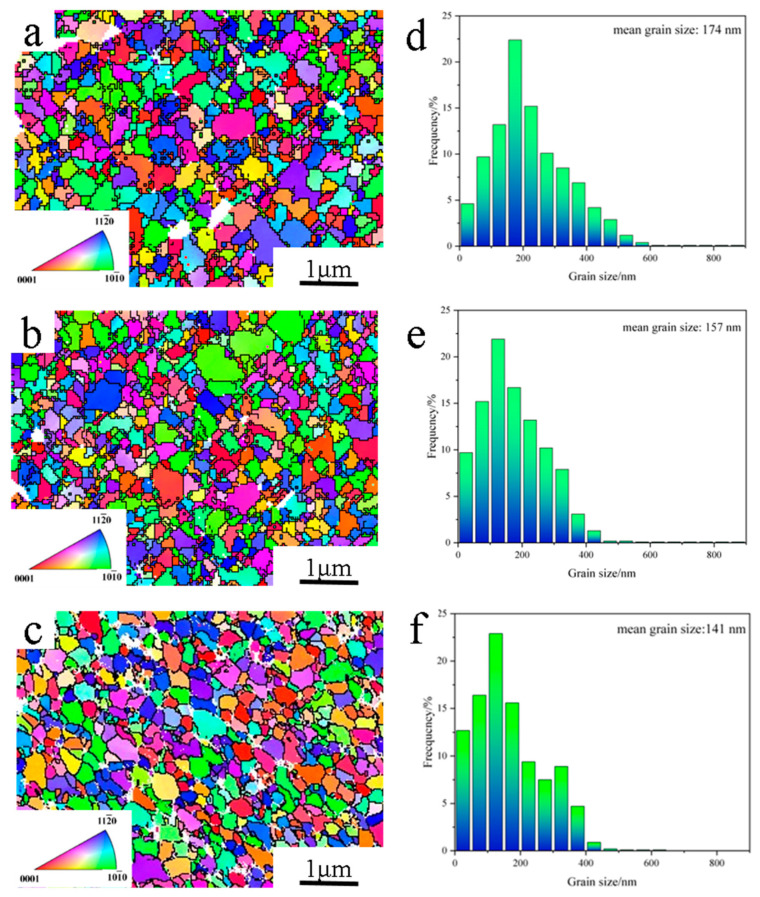
(**a**–**c**) EBSD image and (**d**–**f**) WC grain size distribution of the HPSed WC/Al_0.5_CoCrFeNiTi_0.5_ cemented carbide: (**a**,**d**) 17 wt.% Al_0.5_CoCrFeNiTi_0.5_, (**b**,**e**) 20 wt.% Al_0.5_CoCrFeNiTi_0.5_, (**c**,**f**) 25 wt.% Al_0.5_CoCrFeNiTi_0.5_.

Figure 5 shows the EBSD micrographs and grain size distributions of WC/Al_0.5_CoCrFeNiTi_0.5_ cemented carbide. With increasing content of the Al_0.5_CoCrFeNiTi_0.5_ binder, the WC grain size decreases from 174 nm to 141 nm, indicating that the Al_0.5_CoCrFeNiTi_0.5_ binder helps inhibit the grain growth of the WC grains during HPS. This phenomenon significantly differs from the popular belief for the WC/Co cemented carbide, i.e., the higher the Co binder content in WC/Co cemented carbide, the larger the WC grain size. According to the Austenitic ripening hypothesis and the LSW theory [15,16], the growth of WC grain mainly relies on the “dissolution-precipitation” mechanism where small WC grains dissolve firstly and then will precipitate onto the large undissolved WC grains. Differently, in this study, small WC particles dissolve into W and C atoms and diffuse into the Al_0.5_CoCrFeNiTi_0.5_ binder. As a result, M(Co,Fe,Ni)_3_W_3_C phases are formed by the interaction between the atoms in the binder and the free-stated W and C atoms. With the increase of the Al_0.5_CoCrFeNiTi_0.5_ binder content, more M(Co,Fe,Ni)_3_W_3_C phases are formed, reducing the amount of free-stated W and C atoms and suppressing the growth of WC grain in the WC/Al_0.5_CoCrFeNiTi_0.5_ cemented carbide.

According to the dissolution-precipitation mechanism, the growth of WC grain also closely related to the diffusion process of W and C atoms. HEA has a saturated solid solution degree and is a stable multi-principal solid solution alloy. The solubility of the solid solution is generally determined by atomic size and electronegativity. The atomic radius ($r_{W}=$ 1.37 Å) and electronegativity ($E_{W}$ = 2.36) of W differ significantly from those of the constituent elements in the HEA binder, resulting in a lower solubility of W atoms in the HEA binder [6]. Additionally, the interdiffusion of the atoms in the Al_0.5_CoCrFeNiTi_0.5_ binder in the opposing directions is necessary for the diffusion of W and C atoms. However, the Al_0.5_CoCrFeNiTi_0.5_ binder’s lattice distortion and atom interaction atoms seriously affect the synergistic diffusion between the main elements, leading to the slow mutual diffusion of each element [17,18]. As a result, the back-diffusion of W and C atoms is resisted, which reduces the dissolution and precipitation rate of W and C atoms in the Al_0.5_CoCrFeNiTi_0.5_ binder, thus inhibiting the growth of WC grains.

**Figure 6:**
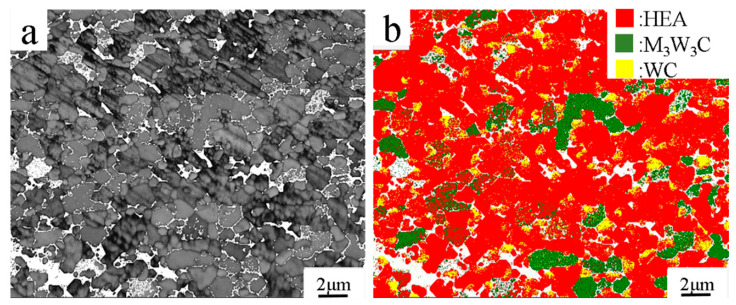
EBSD micrographs of Al_0.5_CoCrFeNiTi_0.5_ binder region in HPSed WC/Al_0.5_CoCrFeNiTi_0.5_ cemented carbide. (**a**) Band contrast; (**b**) Phase map.

Figure 6 shows the band contrast micrograph and phase distribution micrograph of the Al_0.5_CoCrFeNiTi_0.5_ binder region. In the phase map, the Al_0.5_CoCrFeNiTi_0.5_, M_3_W_3_C and WC phase are colored in red, green and yellow, and the content are 68.9%, 14.5% and 5.8% respectively. It is worth pointing out that the proportion of the M(Co,Fe,Ni)_3_W_3_C phase is much higher than that of the WC phase. This further supports the dissolution-precipitation mechanism, i.e., the majority of the W and C atoms react with the elements in Al_0.5_CoCrFeNiTi_0.5_ binder and form the M(Co,Fe,Ni)_3_W_3_C phase firstly, then the retained W and C atoms aggregate and re-precipitate as WC phase. The exitance of WC phase in Al_0.5_CoCrFeNiTi_0.5_ binder region also suggests that the Al_0.5_CoCrFeNiTi_0.5_ binder served as obstacle for the back-diffusion of W and C atoms and re-precipitation onto the large undissolved WC grains, which also helps to restrain WC grain growth.

## 4. Conclusions

In this work, fine-grained WC cemented carbides were prepared by hot-press sintering using 17~25 wt.% Al_0.5_CoCrFeNiTi_0.5_ as binder, and the effects of Al_0.5_CoCrFeNiTi_0.5_ binder on the microstructure characteristics of WC cemented carbide are investigated. The average size of WC grain in the cemented carbides was less than 200 nm, and decreases with the increasing content of Al_0.5_CoCrFeNiTi_0.5_ binder. That was because the small WC particles could decompose into W and C atoms and diffuse into the Al_0.5_CoCrFeNiTi_0.5_ binder during HPS. On one hand, the W and C atoms reacted with the elements in the binder and formed the M(Co, Fe, Ni)_3_W_3_C phase, consuming some C and W atoms. On the other hand, the low diffusion of C and W atoms in Al_0.5_CoCrFeNiTi_0.5_ binder restricted their back-diffusion and re-precipitation onto the large undissolved WC grains. Thus, the Al_0.5_CoCrFeNiTi_0.5_ binder effectively inhibited WC grain growth. This work provides theoretical guidance for the development of new WC/HEA cemented carbide.

## Figures and Tables

**Table 1:** Chemical composition of Al0.5CoCrFeNiTi0.5 HEA.

Element		Al	Cr	Fe	Ni	Ti	Co
Content	wt.%	4.95	19.55	21.43	22.13	8.95	Bal.
	at.%	9.67	19.82	20.23	19.87	9.85	Bal.

## Data Availability

Raw data are available upon request.
